# One Health study of mobile colistin resistance (*mcr*) in *Salmonella enterica* in Canada, 2017–2022

**DOI:** 10.1128/spectrum.02156-24

**Published:** 2025-05-19

**Authors:** Ketna Mistry, David Thumbi, Xiao Rui Li, Audrey Charlebois, Brent P. Avery, Anne E. Deckert, Ashley C. Cormier, Colleen Murphy, Ashley Kearney, Jennifer Campbell, Sara Christianson, David C. Alexander, Sameh El Bailey, Sadjia Bekal, Linda Chui, Xiaofeng Ding, Tanis Dingle, David Haldane, Linda Hoang, Jessica Minion, Samir Patel, George Zahariadis, Celine Nadon, Michael R. Mulvey, Carolee A. Carson, Richard J. Reid-Smith, Amrita Bharat

**Affiliations:** 1 National Microbiology Laboratory Branch, Public Health Agency of Canada 41687, Guelph, Ontario, Canada; 2 National Microbiology Laboratory Branch, Public Health Agency of Canada 41687, Winnipeg, Manitoba, Canada; 3 National Microbiology Laboratory Branch, Public Health Agency of Canada 41687, Saint-Hyacinthe, Québec, Canada; 4 Centre for Food-borne, Environmental and Zoonotic Infectious Diseases, Public Health Agency of Canada 41687, Guelph, Ontario, Canada; 5 Cadham Provinical Laboratory, Diagnostic Services, Shared Health 366539 , Winnipeg, Manitoba, Canada; 6 Medical Microbiology and Infectious Diseases, University of Manitoba 574854, Winnipeg, Manitoba, Canada; 7 Department of Laboratory Medicine, Horizon Health Network 10068, Saint John, New Brunswick, Canada; 8 Département de Bactériologie, Laboratoire de santé publique du Québec 7610 , Sainte-Anne-de-Bellevue, Québec, Canada; 9 Département de Microbiologie, Infectiologie et Immunologie, Université de Montréal 5622, Montréal, Québec, Canada; 10 Alberta Precision Laboratories, Public Health Laboratory 590576 , Edmonton, Alberta, Canada; 11 Department of Laboratory Medicine and Pathology, University of Alberta 536883, Edmonton, Alberta, Canada; 12 Provincial Laboratory Services, Queen Elizabeth Hospital 27381, Charlottetown, Prince Edward Island, Canada; 13 Alberta Precision Laboratories, Public Health Laboratory 590576 , Calgary, Alberta, Canada; 14 Department of Pathology and Laboratory Medicine, University of Calgary 574842, Calgary, Alberta, Canada; 15 Department of Pathology and Laboratory Medicine, Queen Elizabeth II Health Sciences Centre 3686, Halifax, Nova Scotia, Canada; 16 Public Health Laboratory, British Columbia Center for Disease Control, Vancouver, British Columbia, Canada; 17 Saskatchewan Health Authority, Department of Laboratory Medicine, Roy Romanow Provincial Laboratory, Regina, Saskatchewan, Canada; 18 Public Health Ontario Laboratory, Public Health Ontario 97922 , Toronto, Ontario, Canada; 19 Department of Pathology and Laboratory Medicine, Newfoundland and Labrador Public Health and Microbiology Laboratory, St. John’s, Newfoundland and Labrador, Canada; Quest Diagnostics Nichols Institute, Chantilly, Virginia, USA

**Keywords:** antimicrobial resistance, colistin, *Salmonella enterica*, *mcr *genes, mobile colistin resistance, Staramr

## Abstract

Colistin is a last-resort treatment for highly drug-resistant bacterial infections. Of 47,184 *Salmonella* isolates collected from 2017 to 2022 in Canada from human and animal/food sources, mobile colistin resistance (*mcr*) variants conferring colistin resistance were detected exclusively in humans (*n* = 15). These variants were *mcr*-1.1 (*n* = 7), *mcr*-3.1 (*n* = 5), *mcr*-3.2 (*n* = 2), and *mcr*-1.2 (*n* = 1). The most common *mcr*-containing serotypes were I 4,[5],12:i:- (*n* = 8) and Typhimurium (*n* = 3). The proportion of *Salmonella* carrying *mcr* genes remains low in Canada (0.03%).

**IMPORTANCE:**

Colistin can be used in combination with other drugs as salvage therapy for extensively drug-resistant infections. If mobile colistin resistance (*mcr*) becomes widely disseminated in *Enterobacterales*, colistin will no longer be an option for salvage therapy in otherwise untreatable infections. While colistin is not commonly used to treat human *Salmonella* infections, *Salmonella* represents an important reservoir of *mcr* genes that may be transmitted to other gram-negative bacteria. Our aim was to determine the occurrence of *mcr* genes in *Salmonella* isolates collected from humans, food animals, and retail meats in Canada.

## OBSERVATION

As the prevalence of infections with multidrug-resistant (MDR) and extensively drug-resistant (XDR) *Enterobacterales* increases, colistin remains a last-resort option for these difficult-to-treat human infections. The World Health Organization’s AWaRe (Access, Watch, and Reserve) categorization of antimicrobials lists colistin in the “Reserve” category: antimicrobials that should be reserved for treatment of confirmed or suspected infections with MDR organisms (1). Colistin has limited efficacy as monotherapy and can cause significant side effects; however, it is used in combination with other drugs as salvage therapy for extensively drug-resistant infections. If mobile colistin resistance (*mcr*) becomes widely disseminated in *Enterobacterales*, colistin will no longer be an option for salvage therapy in otherwise untreatable infections. For example, pan-resistant *Klebsiella pneumoniae* with resistance to carbapenems, tigecycline, and colistin have been reported (2, 3). The most common use of colistin is in combination therapy of infections caused by carbapenemase-producing *Enterobacterales* (CPE). The incidence of hospital-associated CPE in Canada was 0.14 infections per 10,000 patient days in 2022 (4). The United States Centers for Disease Control’s Multi-site Gram-negative Surveillance Initiative Report from 2021 identified an overall incidence of carbapenem-resistant *Enterobacterales* (CRE) of 6.1 cases per 100,000 population (https://www.cdc.gov/hai/eip/pdf/mugsi/2021-CRE-Report-508.pdf). A recent global study found an overall resistance rate of 4.5% for CRE in 2019 (5). All isolates in our study were susceptible to meropenem. While colistin is not commonly used to treat human *Salmonella* infections, *Salmonella* represents an important reservoir of *mcr* genes that may be transmitted to other gram-negative pathogens (6). A global study of *mcr* genes in 2018 found that *mcr* was distributed among *Escherichia coli* (411), *Salmonella enterica* (29), *Klebsiella pneumoniae* (8), *Escherichia fergusonii* (2), *Kluyvera ascorbata* (2), *Citrobacter braakii* (2), *Cronobacter sakazakii* (1), and *Klebsiella aerogenes* (1) (7). Many studies have shown that diverse *mcr* plasmids can undergo horizontal transfer via conjugation, including transfer of *mcr* between *Salmonella* and *E. coli* (7–10).

Acquired colistin resistance occurs by one of two known pathways, chromosomally mediated resistance mutations (e.g., in the *pmrAB* and/or *phoPQ* genes) or plasmid-mediated *mcr* genes (11). Colistin is a cationic polypeptide that disrupts the outer membrane by binding to lipopolysaccharide (LPS). The *mcr* gene encodes a phosphatidylethanolamine lipid A transferase which modifies the LPS by adding a phosphatidylethanolamine group to the lipid A moiety (11–14). This reduces the net negative charge of the membrane and leads to reduced binding of colistin.

The first *mcr* gene reported was *mcr*-1 from an *Escherichia coli* isolate in China in 2015 (15), and this allele has now disseminated worldwide (7). Following this initial report, a retrospective analysis of 1,600 whole-genome sequences of *Enterobacterales* from Canada found three occurrences of *mcr*-1 in *E. coli* (16). Subsequently, an *mcr-*3.2 variant in *Salmonella* was identified in Canada from a patient who had travelled to Thailand (17). Currently, there are 10 *mcr* alleles described in the literature (*mcr*-1 to *mcr*-10). Most alleles have additional variants, e.g., mcr-1.1 (18, 19), and some, like *mcr*-9, do not confer colistin resistance in *Enterobacterales* (20, 21). Colistin (polymyxin E) and polymyxin B have been used globally in veterinary medicine, and in some countries, colistin has been used as a growth promoter administered in animal feed (22, 23). Health Canada lists colistin as a Category I or “Very High Importance” antimicrobial for human medicine, and it is not approved for sale for use in animals in Canada (https://www.canada.ca/en/health-canada/services/drugs-health-products/veterinary-drugs/antimicrobial-resistance/categorization-antimicrobial-drugs-based-importance-human-medicine.html). Our aim was to determine the occurrence of *mcr* genes in *Salmonella* isolates collected from humans, food animals, and retail meats in Canada.

The Canadian Integrated Program for Antimicrobial Resistance Surveillance collected 47,184 *Salmonella* isolates between 2017 and 2022 from humans (*n* = 33,579), food animals (*n* = 11,468), and meat obtained from retail stores (*n* = 2,137). The food and animal component included veterinary diagnostic clinical isolates (sick animals do not enter the food chain), as well as healthy animals on farms and at abattoirs. PulseNet Canada and the National Microbiology Laboratory Branch’s Guelph Reference Services Unit carried out whole-genome sequencing using Illumina Nextera XT library preparation kits on Illumina MiSeq or NextSeq platforms (MiSeq v3 600 cycle/NextSeq v2.5 Mid Output 300 cycle kits). The Staramr program (v0.9.1) was used in the Galaxy platform (24) to detect antimicrobial resistance genes and mutations and to predict resistance phenotypes. *Salmonella* serotyping was completed using the *Salmonella In Silico* Typing Resource (SISTR v1.1.1) (25). Antimicrobial susceptibility testing was performed by broth microdilution on the Sensititre ARIS System (Thermo Fisher Scientific, Waltham, Massachusetts). The Clinical and Laboratory Standards Institute (CLSI) does not provide colistin breakpoints for *Salmonella* species. Minimum inhibitory concentrations (MICs) were interpreted using CLSI breakpoints for other *Enterobacterales*, with colistin resistance defined as MIC >= 4 mg/L (26). PulseNet Canada uploads whole-genome sequencing data for *Salmonella* to the NCBI SRA database under BioProject PRJNA543337.

**Fig 1:**
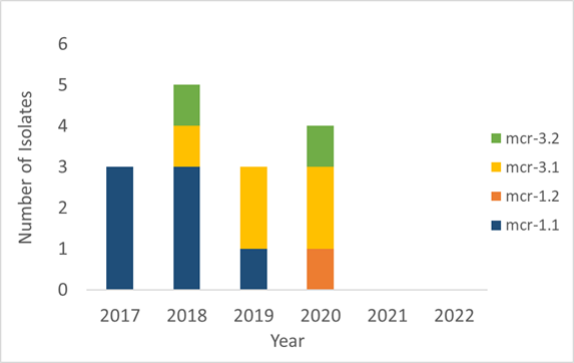
Distribution of mobile colistin resistance alleles identified in *Salmonella* from 2017 to 2022. Detection of *mcr* genes was carried out on 47,184 *Salmonella* from human and nonhuman sources using the staramr program. Fifteen human-sourced isolates carried an *mcr* allele that confers colistin resistance. The number of isolates identified in each year and the specific *mcr* alleles are shown.

Among 13,605 animal/food-source *Salmonella* screened, we only detected *mcr* alleles that did not confer colistin resistance: *mcr*-9 (*n* = 8) and *mcr*-4.3 (*n* = 1). MICs ranged from <= 0.25 to 0.5 mg/L, and others have similarly reported that these alleles do not confer resistance (20, 21, 27). Among 33,759 human-source *Salmonella*, we identified 15 isolates from 15 different human hosts (0.03%) with *mcr* alleles that predict phenotypic resistance to colistin, including *mcr*-1.1 (*n* = 7), *mcr*-3.1 (*n* = 5), *mcr*-3.2 (*n* = 2), and *mcr*-1.2 (*n* = 1) (Fig. 1). Of the 12 isolates available for phenotypic testing, all were resistant to colistin with MICs >= 8 mg/L. The three isolates that were not available for MIC testing contained *mcr*-1.2 (*n* = 1) and *mcr*-3.1 (*n* = 2), alleles that have been shown to confer colistin resistance (28, 29).

Within these 15 isolates, 13 (87%) were from stool, and two (13%) were from urine. Of the 14 isolates with available sex of the host, 8 (53%) were from males. Host ages ranged from 1 to 46 years old. *Salmonella* serotypes I 4,[5],12:i:- (*n* = 8) and Typhimurium (*n* = 3) accounted for 73% of the serotypes with colistin resistance. Other serotypes included *S*. Agona (*n* = 1), *S*. Cerro (*n* = 1), *S*. Enteritidis (*n* = 1), and *S*. Paratyphi B variant Java monophasic (*n* = 1). Our results were similar to a global study on *mcr*-containing *S. enterica*, which also found that the most common serotypes were Typhimurium and I 4,[5],12:i:-, and humans were the predominant source (30).

The *mcr*-4 variant identified in our analysis, *mcr*-4.3 (found in *S*. Mbandaka), did not confer resistance to colistin (MIC <= 0.25 mg/L). A study from the Netherlands similarly found that isolates with *mcr*-4.3 were susceptible, while those with *mcr*-4.6 were resistant. They identified three human source *E. Kobei* isolates harboring *mcr*-4.3 that were susceptible to colistin (MIC >= 1 mg/L) and three livestock isolates, including one *E. coli* and two *H*. *paralvei*, harboring *mcr*-4.6 that were resistant (MIC 4–8 mg/L) (27). Curiously, when *mcr*-4.3 and *mcr*-4.6 were transformed into *Acinetobacter baumannii*, resistance was not observed. These observations suggest that whether an *mcr* allele confers resistance may depend on the species or serotype, particularly for *mcr*-4 variants (27, 31).

We observed that *Salmonella* carrying *mcr*-9 was first detected in Canada in 2022 (*n* = 15 isolates; seven from humans and eight from animal/food). Other studies have reported that *mcr*-9 does not confer colistin resistance in *Salmonella* and *E. coli* (20, 21). One study evaluated over 100 *Salmonella enterica* and *Escherichia coli* isolates with *mcr*-9 and found that all isolates were susceptible to colistin (21). Similarly, we found that all 15 Canadian isolates with *mcr*-9 did not confer resistance to colistin. In 2021 and 2022 (the last 2 years of this study), *mcr* conferring colistin resistance was not detected from any human or animal/food *Salmonella* (*n* = 12,506 isolates). This highlights the importance of continued antimicrobial stewardship.

In summary, all isolates in this study with *mcr* alleles conferring colistin resistance were from humans, and the most common alleles were *mcr*-1.1 and *mcr*-3.1. Overall, mobile colistin resistance remains uncommon in Canada. We found that the presence of the *mcr*-4 gene variant, *mcr*-4.3, did not confer resistance to colistin. Continued surveillance of acquired colistin resistance, including emerging variants and phenotypes, plays an important role in monitoring the potential threat of colistin resistance.

## Data Availability

PulseNet Canada uploads whole-genome sequencing data for *Salmonella* to the NCBI SRA database under BioProject PRJNA543337.
